# Thymidine Phosphorylase Expression and Microvascular Density Correlation Analysis in Canine Mammary Tumor: Possible Prognostic Factor in Breast Cancer

**DOI:** 10.3389/fvets.2019.00368

**Published:** 2019-10-25

**Authors:** Nicola Zizzo, Giuseppe Passantino, Roberta Maria D'alessio, Antonella Tinelli, Giuseppe Lopresti, Rosa Patruno, Domenico Tricarico, Fatima Maqoud, Rosa Scala, Francesco Alfredo Zito, Girolamo Ranieri

**Affiliations:** ^1^Section of Veterinary Pathology and Comparative Oncology, Department of Veterinary Medicine, University of Bari “Aldo Moro”, Valenzano, Italy; ^2^MD Freelancer, Bristol, United Kingdom; ^3^Section of Pharmacology, Department of Pharmacy-Pharmaceutical Sciences, University of Bari, Bari, Italy; ^4^Interventional and Medical Oncology Unit, Department of Pathology National Cancer Research Centre, IRCCS Istituto Tumori Giovanni Paolo II, Bari, Italy

**Keywords:** canine mammary tumor, thymidine phosphorylase, histopathology, microvasculature, cell proliferation

## Abstract

**Purpose:** The thymidine phosphorylase (TP) is a key enzyme involved in the metabolism of pyrimidines. Inhibition or downregulation of this enzyme causes accumulation of metabolites with consequences in DNA replication. TP regulates angiogenesis and chemotactic activity of endothelial cells. Different studies showed the presence of TP upregulation in human cancer but the correlation between TP expression and the microvascular density (MVD) in canine mammary tumors is unknown. The aim of this study was to investigate a possible correlation between the MVD and TP expression in tumor cells of canine mammary tumors of different degree of severity (G1–G3) by immunohistochemical analysis.

**Methods:** Sixty-eight samples of spontaneous mammary neoplasia of 5–12 cm in diameter were collected from purebred and mixed-breed dogs (mean aged = 9.5 ± 7), not subject to chemotherapy treatments in veterinary clinics. Histopathological analysis and immunostaining were performed.

**Results:** Carcinoma simple samples have been classified as 72.06% of tubule-papillary, 20.59% cysto-papillary, and 7.35% tubular carcinomas. Immunostainings revealed a marked cytoplasmic expression of TP in 30.88% of samples, mild in 32.35%, weaker in 22.07%, and negative in 14.70%. The correlation analysis and two-way ANOVA showed a linear correlation between MVD and TP with a coefficient of correlation (*r*) > 0.5 (*p* < 0.05) in G2 and G3. No correlation between variables was found in G1.

**Conclusions:** These findings suggest that cytoplasmic TP overexpression is correlated with microvascular density in canine mammary tumors, in severe grade, and it can be a potential prognostic factor in breast cancer.

## Introduction

The thymidine phosphorylase (TP) enzyme was purified in the mid-1970s from both *Escherichia coli* and *Salmonella* spp. (1). This enzyme is encoded by a gene of chromosome 22, in position 13 of the long arm (22q13) and consists of two subunits of 47 kDa in eukaryotic cells (45 kDa in *Salmonella* spp.). The TP is involved in the metabolism of pyrimidines that catalyzed the reversible reaction: thymidine or deoxyuridine + orthophosphate ↔ thymine or uracil + 2-deoxy-D-ribose 1-phosphate (2–4). TP activity reduction causes an accumulation of thymidine (dThd) and deoxyuridine (dUrd) in blood and tissues, causing an imbalance in the nucleotide pool. As a consequence, the mitochondrial DNA becomes abnormal and presents point mutations, multiple deletions, and depletion (5). TP is considered to be a homolog of the endothelial platelet factor (PD-ECGF), its function is promoting angiogenesis and the chemotactic activity of endothelial cells (2, 6).

The enzyme TP is physiologically present in the cytoplasm of platelets, in the glandular and stromal epithelium during the female menstrual cycle (7–9), moreover its presence has been pathologically highlighted in chronic inflammations such as rheumatoid arthritis, arthrosis, psoriasis (10, 11).

Several studies have shown that TP upregulation induces the oncogene Pi3 kinase/Akt pathway, inhibits the autophagic BNIP3 gene and the apoptotic caspases 3/9 pathway with an anti-apoptotic action promoting proliferation (6, 12). The BNIP3, caspases 3/9 and Pi3 kinase/Akt pathways are involved in several repairing function in the tissues other than cancer (13, 14). TP is pathologically overexpressed in several human cancers and it is reported to be associated with poor outcome. For instance, TP gene has been found overexpressed in hepatic, gastric and mammary tumor, oral squamous carcinoma, bladder, and prostate cancers (15–17), meanwhile, TP related protein has been detected in the plasma of subjects with neoplasia (18). Several studies have shown that overexpression of TP may have a predictive role in women's breast cancer (19, 20), and this is linked to the carcinogenesis process. Indeed, mast cell expresses a cytoplasmic and/or nuclear immunohistochemical reactivity, with an increase of microvascular density in epithelial, endothelial, macrophage tumor microenvironment (15, 21).

For this purpose, a commercial TP inhibitor, a tipiracil/trifluridine based drug, is now available for clinical use. This drug is indicated for the treatment of metastatic colorectal cancer in patients previously treated with fluoropyrimidine-, oxaliplatin-, and irinotecan-based chemotherapy, anti-VEGF biological therapy and anti-EGFR therapy in case of RAS positivity.

Accumulating evidences suggest that TP upregulation is associated with a beneficial response to chemotherapy. TP gene has been shown to be upregulated by docetaxel, paclitaxel, cyclophosphamide, and oxaliplatin through the induction of inflammatory cytokines (18, 22). Capecitabine, which is largely used for metastatic breast cancer, is a prodrug converted to the active drug 5-fluorouracil by the elevated expression levels of thymidine phosphorylase. The upregulation of the TP gene by histone deacetylase inhibitors potentiates the capecitabine action resulting in synergistic/ additive antiproliferative and pro-apoptotic effects in metastatic breast cancer cells (23). Bevacizumab potentiates the anti-tumor effects of 5-FU in colon cancer xenograft mice and increase 5-FU concentration in tumors by up-regulating thymidine phosphorylase (TP) in colon cancer (24).

Considering that the spontaneous mammary tumors of the dogs have a biological and histopathological behavior similar to that of the woman, and that the human TP shares 39% of identity with the prokaryotic one (25), we investigated the role of TP in the canine mammary tumor cells highlighting the immunohistochemical expression patterns correlating them to tissue neovascularization (microvascular density -MVD) and tumor grade/stage. Transcriptome gene analysis showed the presence of a common gene in canine mammary tumor and woman's breast cancers (26). Currently, no data are available on the role of TP gene in canine mammary tumors. This type of tumor in dogs is under-investigated despite the potential relevance of this spontaneous tumor. The mammary cancer in dog is indeed under the same hormonals and environmental stimulus as the woman breast cancer.

Although, previous works, investigated the endothelial area and microvascular density in a canine non-Hodgkin's lymphoma showing that this can be used an as interspecies model of tumor angiogenesis (27, 28).

## Materials and Methods

A total of 68 samples of spontaneous mammary neoplasia of 5–12 cm in diameter were collected from purebred and mixed- breed dogs aged between 4 and 15 years (average 9.5), not subject to chemotherapy treatments, from 2015 to 2019 in various veterinary clinics in Bari, Italy. The collected samples were surgically removed from female dogs by radical mastectomy or by regional mastectomy, with or without removal of the inguinal lymph node. All the collected samples did not show the presence of metastasis.

Surgical samples were fixed in 10% buffered formalin and stained for the histological diagnosis with Hematoxylin-Eosin. Over a total of 68 samples of carcinoma simple (CS) were characterized and classified following Goldschmidt classification (2011) (29). The histological gradation was determined according to the evaluation of three morphological characteristics: (i) degree of glandular differentiation assessed through tubular formation; (ii) nuclear pleomorphism; (iii) mitotic activity. Each parameter has been classified into three categories, each with a score of 1–3. The scores of all three components were added together for a total of 3–9 points. The grade was assigned an arbitrary division as follows: degree 1 (G1), well-differentiated or low grade: 3–5 points; grade 2 (G2), moderately differentiated or intermediate degree: 6–7 points, and grade 3 (G3), poorly differentiated or high grade: 8–9 points (30–33). Neoplastic sections have been immunohistochemically stained according to the labeled streptavidin avidin-biotin (LSAB) method (19). Tissue sections were cut (4 μm thick), placed on poly-L-lysine-coated glass slides, and, subsequently, deparaffinized in xylene and dehydrated. To detect the thymidine phosphorylase and endothelial markers, the sections were immersed in citrate buffer (0.1, pH 0.6) and subjected to microwave irradiation for 15 min. All the sections were treated for 30 min with 0.3% hydrogen peroxide, then in methanol for 12 min to quench endogenous peroxidase activity (12 ml H_2_O_2_ in 400 ml of methanol). After washing three times for 5 min each with phosphate buffered saline (PBS), the sections were blocked by soaking for 20 min at room temperature in PBS containing 1% bovine serum albumin. The blocked sections were incubated overnight at 48°C anti-mouse primary antibodies for TP PD-ECGF AB1 (clone PGF 44.C) (Neo Markes, Freemount, California, USA, diluted 1/100) (15) and monoclonal mouse antibody anti-CD31 Clone JC70A (Dako, Glostrup, Denmark) diluted 1/50, for 40 min then thoroughly washed in 0.05 M buffered with Tris saline (pH = 7.6) and incubated with streptavidin-peroxidase (Dako, 1: 100) for 40 min. 3, 3- diaminobenzidine (DAB) (Dako Glostrup, Denmark) was used as the chromogen; to counteract the core of Gill'S hematoxylin (Polysciences, Warrington, PA, USA) after sections have been dehydrated and assembled (34, 35). We do also have evidence of negative controls not immunostained and positive control of not tumor cells from canine expressing TP that show sporadic area of immunostaining. The histologic evaluation was performed independently by 2 of the authors (NZ and GP). The sections were examined at first with a magnification of × 200 (i.e., × 20 objective and × 10 ocular lens; 0.7386 mm^2^ per field). Subsequently the cell count has been performed at × 400 × field (i.e., × 40 objective and × 10 ocular lens; 0.1885 mm^2^ for field). Starting the count from the top right of all the colored sections, moving down and to the left. The immunostaining of 50 cells was evaluated × field (total number of immunostained cells = 500 cells × 10 fields) per sample. According to Soini et al. the intensity of the immunostaining with the TP antibodies were evaluated by dividing the staining reactions of cytoplasmic cells and nucleus into four groups (36) (Table 1). The quantitative evaluation of the immunostaining was also defined (Table 2). A combined score for the immunostaining, based on both qualitative and quantitative immunostaining, was composed by adding together the qualitative and quantitative scores. The combined scores were then divided into four main groups (Table 3). The level of TP expression was defined as positive if the predominant intensity was 2 or 3, negative if the predominant intensity was 0 or 1.

**Table 1:** Classification: immunostaining of cytoplasmic cells and nucleus of tumor cells.

**Group**	**Staining intensity**	**Intensity (%)**
1	Weak	<25%
2	Moderate	25–50%
3	Strong	50–75%
4	Very strong	75–100%

**Table 2:** Classification: percentage of tumor cells showing cytoplasmic positivity.

**Group**	**Positivity (%)**
0	No positive immunostaining
1	<25%
2	25–50%
3	50–75%
4	>75%

**Table 3:** Classification: immunostaining degree Quanti-Qualitative.

**Group**	**Immunostaining**	**Scores**
–	Negative	0
+	Moderate	1
++	Strong	2
+++	Very strong	3

The microvessel density (MVD) and endothelial area (EA) found at least at 1 mm from the edge of the tumor, were evaluated on 10 stromal areas counting the number of single vessels per field or groups of cells with or without lumen in hot-spot areas with the highest vascularization and positively stained of fields ×400 magnification (19, 34).

The number of immunostained cells were counted and plotted vs. frequency of finding cells × field.

### Statistical Analysis

An image analysis system was used to evaluated MVD, EA, and TP. Average results ±S.D. were evaluated for all samples and statistical analysis. The association between TP expression and the histological degree were evaluated using *T*-Test student. MVD was evaluated in the area with the highest vascularization according to Weidner et al. (37).

Single or groups of endothelial cells and micro-vessel in the stroma of tumor cells and in CS have been evaluated as EA and MVD. MVD and EA were determined for each CS and subgroups based on the histological malignant. The meaning of the difference between MVD, EA, and TP between the simple carcinoma was evaluated with *t*-Test Student, Pearson correlation coefficient and two ways ANOVA. Superior Performance Software System (SPSS) software, R software, A Language and Environment for Statistical Computing were used to perform all the statistical analysis.

## Results

**Figure 1:**
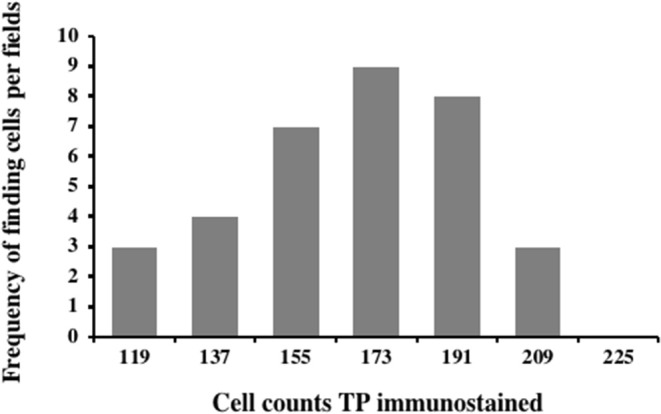
Frequency-distribution of the cell TP immunostained per field from 44 samples corresponding to G2 and G3. A normal distribution was observed in the range between 103 and 205 of TP immunostained cells with a mean of 153.35 ± 4.8, median: 155.5, variance: 790.53, and class value of 16.7.

**Figure 2:**
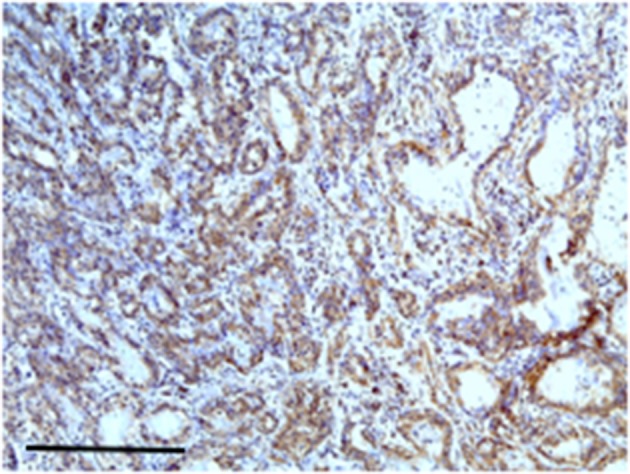
The figure shows the immunohistochemical reactivity with primary antibodies against TP located in the cytoplasm of the neoplastic mammary epithelial cells, nucleus was immunostained but at lower intensity. Bar 100 μm.

**Figure 3:**
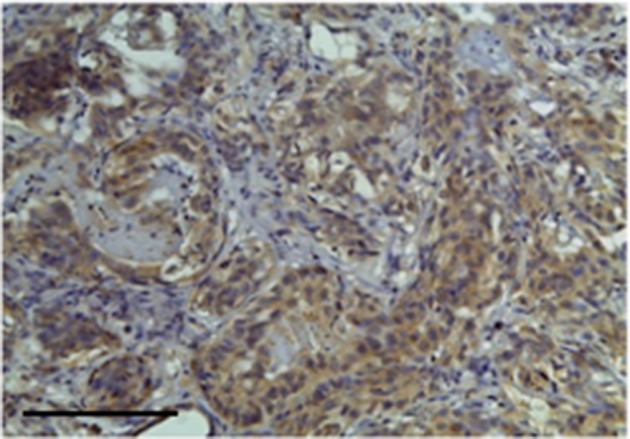
The figure shows the immunohistochemical reactivity classified as very strong immunostaining primary antibodies against TP located in the cytoplasm of the neoplastic mammary epithelial cells, Bar 100 μm.

**Figure 4:**
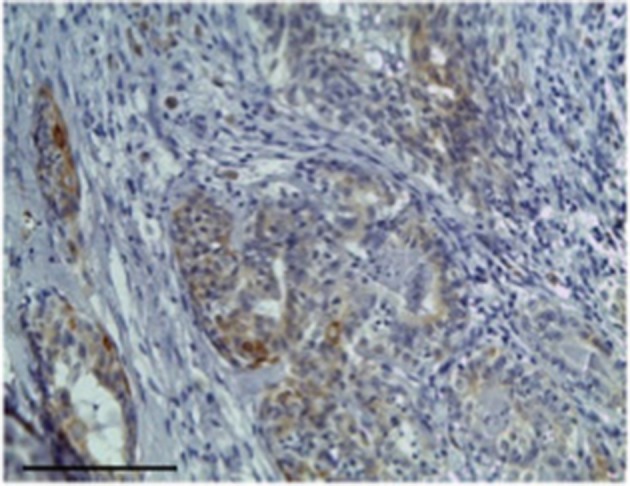
The figure shows the immunohistochemical reactivity classified as strong immunostaining primary antibodies against TP located in the cytoplasm of the neoplastic mammary epithelial cells, Bar 100 μm.

Carcinoma simple types samples distribution have been reported (Table 4). In relation to the grade, samples were also subdivided (Table 5). The carcinoma–tubulopapillary represented the most frequent form identified followed by the carcinoma–cystic-papillary. In our samples, the carcinoma–tubulopapillary was the most frequent G3 form found which actually represents 66.7% of Grade 3. The frequency-distribution of the number of TP immunostained cells in G2 and G3 were described by a bell-shaped Gaussian distribution curve (Figure 1). TP immunohistochemical reactivity was detected in the cytoplasm of tumor cells of the neoplastic mammary epithelial cells (Figure 2). Nucleus was also immunostained but at lower intensity. According to the intensity of the cytoplasmic reaction, the classification system adopted showed a range from 0 to 3, which includes positives TP cells (from 2 to 3). Immunostainings revealed a marked expression in 21 over 68 (30.88%) (Figure 3), strong in 22 over 68 (32.35%) (Figure 4), weaker in 15 over 68 (22.07%), or negative in 10 over 68 (14.70%) (Table 6).

**Table 4:** Percentage of subtypes of Carcinoma-Simple type.

**Carcinoma–Simple types**	**Total number**	**Percentage**
Carcinoma–Tubulopapillary	50	73.5%
Carcinoma–Tubular	5	7.4%
Carcinoma–Cystic-papillary	13	19.1%
Total	68	100

**Table 5:** Carcinoma-Simple types gradation.

**Histological type**	**Grade 1**	**Grade 2**	**Grade 3**	**Total**
Carcinoma–Tubulopapillary	21 (42.00%)	19 (38.00%)	10 (20.00%)	50 (73.5%)
Carcinoma–Tubular	1 (20.00%)	2 (40.00%)	2 (40.00%)	5 (7.4%)
Carcinoma–Cystic-papillary	2 (15.38%)	8 (61.53%)	3 (23.08%)	13 (19.1%)
Total	24 (35.29%)	29 (42.64%)	15 (22.06)	68 (100%)

**Table 6:** Cytoplasmic immunostaining in different grades.

**Cytoplasmic immunostaining**	**Grade 1**	**Grade 2**	**Grade 3**	**Total**
0	8	2	0	10 (14.70%)
1	7	7	1	15 (22.06%)
2	4	13	5	22 (32.35%)
3	5	7	9	21 (30.88%)
Total	24 (35.29%)	29 (42.11%)	15 (22.08%)	68 (100%)

**Figure 5:**
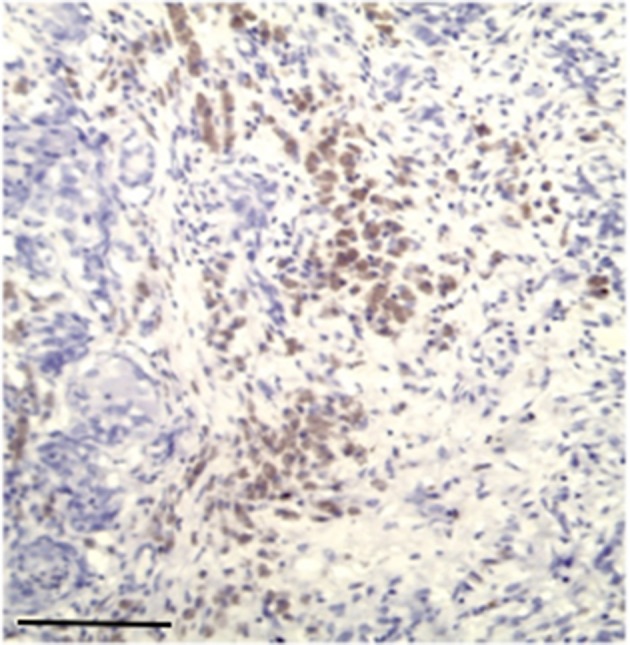
Modest immunostaining positive inflammatory cell with primary antibodies for TP, Bar 100 μm.

**Figure 6:**
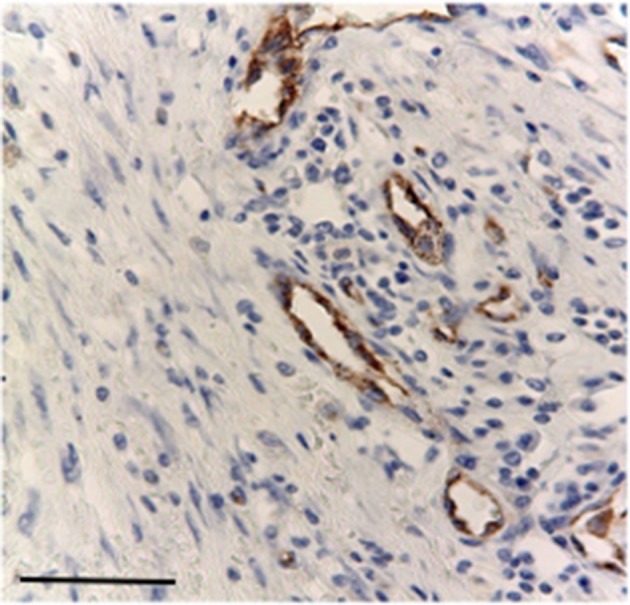
Immunostaining primary antibodies for CD31 in endothelial cells stroma, Bar 100 μm.

Nuclear TP expression was modest or absent and statistically not significant, but it was present in inflammatory cells components and in blood vessels of the stroma (Figures 5, 6). The endothelial cells resulted to be positive and immunostained with CD31.

Mean MVD, EA, and TP on the analyzed sections have been grouped according to the degree (30), in the G3 at X 400 showing a significant difference.

**Figure 7:**
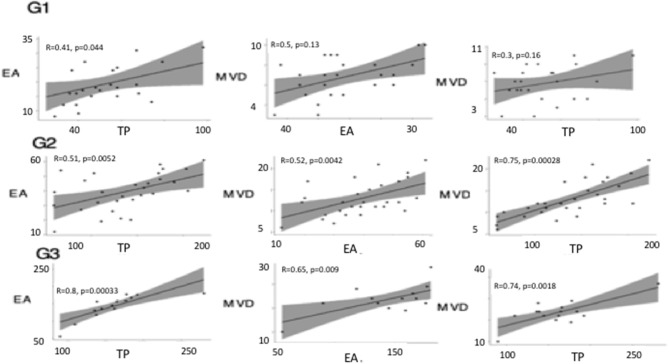
Linear correlation plots between thymidine phosphorylase (TP), micro-vessel density (MVD), and endothelial area (EA) performed in different tumor grade (G1, G2, G3). Linear correlation analysis and two-way ANOVA showed between TP and EA, and, MVD and TP, and MVD and EA a coefficient of correlation (r) > 0.5 (*p* < 0.05) in G3. A significant correlation was calculated between variables in G2.

Linear correlation analysis and two-way ANOVA showed between TP and EA, and MVD and TP a coefficient of correlation (*r*) > 0.5 (*p* < 0.05) in G3, instead, between MVD and EA the coefficient of correlation, was (*r*) > 0.5 with a *p*-value > 0.05. Similarly, a significant correlation was calculated between variables in G2 (Figure 7). In G1 no correlation between variables was calculated.

## Discussion

Our study has outlined that Thymidine Phosphorylase is over-expressed immunohistochemically in canine malignant mammary tumors. The TP over- expression at the histological grade (G3) is correlated to the neo-angiogenesis and with the inflammatory cells in woman (19–21). TP enzyme is indeed found in G3 in human tissues, the highest degree of tumors. Lee et al. suggest a variety of roles for TP enzyme in tumor cell metabolism (21, 38). According to Yang, the Thymidine Phosphorylase might be expressed in macrophages of the tumor stroma, that can release angiogenetic factors with paracrine and autocrine mechanism (20). This mechanism may promote MVD and EA with the tumor progression. However, some authors suggested TP can act as a chemotactic factor but no mitogenic factor for the endothelium (21, 39). The data that we obtained in our study may indicate a role of TP in the proliferative kinetics and pathogenesis of the canine mammary neoplasia, because it could increase the “pool of pyrimidines” available for the transformation of the thymidine 2 deso-oxyribose, an intracellular mediator inducing the production of metalloproteases, VEGF and IL 8 leading to angiogenic effects. In addition, the enzyme recognizes a large number of 5-substituted 2-deoxyuridines with antiviral and/or antitumor activity and the enzyme can deactivate analogous nucleosides such as 5-trifluorotimidine (TFT) which is used as an antitumor compound (23). In our study, the correlation existing within the tumor grade in G2 and G3, the microvascular density and the TP expression by immuno-histological localization strongly supports the idea that TP is a biomarker of cancer progression in the mammary tumor in dogs.

TP can be a valid predictive biomarker for oral fluoropyrimidines like capecitabine in human but not for 5-FU (40–43). The current data are not sufficient to treat patients based on their TP expression status when being considered for fluoropyrimidine chemotherapy. Considering that female dogs develop spontaneous tumors with biological and histopathological behavior similar to human symptoms, they represent a valid animal model for testing new chemotherapeutic agents in “*ex vivo*” experiments and to produce translation data to humans.

The correlation found between grade and TP-expression and microvascular density in addition to the traditional grading could speed up the grading process, leading to an easier and safe immunohistochemistry.

The availability of tissues from spontaneous tumors will allow investigating the effectiveness of new possible combinations of drugs acting on TP-coupled signal. Indeed, TP suppressed the autophagic gene BNIP3 and the caspase 3/9 leading to an anti- autophagic and anti-apoptotic action as well as proliferative effects (6, 12). TP induces the oncogene Pi3 Kinase/Akt which is coupled to the activation of large-conductance potassium calcium channels and Transient Receptor Potential Vanilloid 1 (TRPV1) known to be emerging pharmacological targets in cancer (12, 44–46). The antiproliferative and apoptotic effects of 5-FU are potentiated by the activation of TRPV1 channel into a cellular line of mammary carcinoma (47).

**Figure 8:**
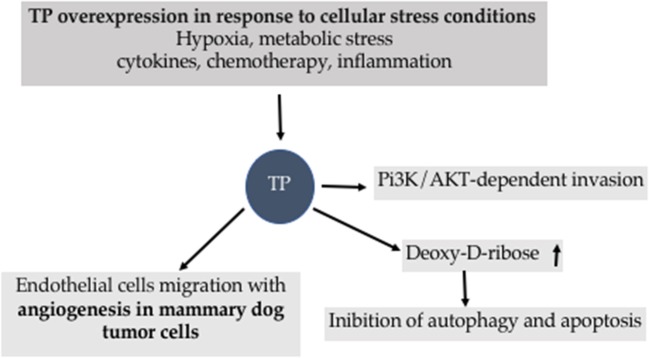
Biological effects of Thymidine Phosphorylase (TP).

TP is under the control of several stimulus and mediates cascade of reactions associated to apoptosis and autophagy (Figure 8).

In conclusion, the elevated incidence of tumors, the large body surface, responses similar to cytotoxic drugs, the short average lifespan, the large volume of distribution are factors which contribute to make the dog an interesting model for future preclinic surveys. Last but not least, humans and animals share the same environment and are therefore subject to the same risk factors that cannot be found in small laboratory animals. The dog, therefore, may help to identify environmental risk factors for human health and can be used as a spontaneous model of study for various types of cancers in humans. In addition, the most common canine breast cancers are positive to estrogen and progesterone receptors as well as to *c-erb*B (48).

Comparing the data in our possession with the data in the literature on human breast cancer, we can highlight a correlation between these two species. As observed in woman breast cancer we found a high degree of TP expression in G2 and G3.

## Data Availability Statement

The datasets generated for this study are available on request to the corresponding author.

## Ethics Statement

This study was carried out in accordance with the recommendations of local clinical veterinary facilities of the University of Bari, Italy. The owners of the animals gave the written informed consent to the use of the sample for experimental purposes other than diagnosis. The hospital admission of the dogs in the veterinary clinics was for surgery and medical treatment. Written informed consent was obtained from the owners for the participation of their animals in this study.

## Author Contributions

NZ and GR: conceptualization, reagents, supervision, and funding acquisition. GP, RP, RD, and AT: methodology. FM and RS: software. DT, NZ, and GR: validation. NZ, GP, RP, RD, and AT: investigation. All the co-authors: writing—review and editing. FM, RS, and DT: visualization. GR: project administration.

### Conflict of Interest

The authors declare that the research was conducted in the absence of any commercial or financial relationships that could be construed as a potential conflict of interest.
